# Antioxidant Potential of Tamarillo Fruits—Chemical and Infrared Spectroscopy Analysis

**DOI:** 10.3390/antiox12020536

**Published:** 2023-02-20

**Authors:** Miguel Rito, Joana Marques, Ricardo M. F. da Costa, Sandra Correia, Tércia Lopes, Daniel Martin, Jorge M. P. L. Canhoto, Luís A. E. Batista de Carvalho, Maria Paula M. Marques

**Affiliations:** 1 Centre for Functional Ecology, Associate Laboratory Terra, Department of Life Sciences, University of Coimbra, Calçada Martim de Freitas, 3000-456 Coimbra, Portugal; 2 Molecular Physical-Chemistry R&D Unit, Department of Chemistry, University of Coimbra, 3004-535 Coimbra, Portugal; 3 InnovPlantProtect CoLab, Estrada de Gil Vaz, 7351-901 Elvas, Portugal; 4 Department of Life Sciences, University of Coimbra, Calçada Martim de Freitas, 3000-456 Coimbra, Portugal

**Keywords:** antioxidant activity, fruit extract, FTIR-ATR, phenols, tamarillo

## Abstract

Native to South America, tamarillo (*Solanum betaceum* Cav.) is a small tree cultivated as a fruit crop in several regions of the world. Known for its sweet and sour taste, tamarillo fruits are very nutritious due to the presence of health-beneficial components such as fiber, vitamins, and antioxidants. Despite its nutritional value, tamarillo remains poorly known in global markets. The present work aims to study the antioxidant activity of four genotypes of tamarillo. Several chemical assays were performed to assess the antioxidant components and antioxidant activity of aqueous ethanolic extracts from each genotype. Overall, the Mealhada genotype (a red cultivar) showed the most interesting results, displaying the highest amount of total phenolic, flavonoids, and anthocyanin contents, as well as higher antioxidant activity. To evaluate the composition of the extract, Fourier-transform infrared spectroscopy (FTIR) was used to characterize important components in aqueous ethanolic extracts of the fruits, having revealed the presence of high amounts of phenols (the main compounds responsible for antioxidant activity), as well as triterpenoids and polysaccharides. The present results highlight the potential nutraceutical importance of tamarillo fruits.

## 1. Introduction

Tamarillo (*Solanum betaceum* Cav.) is a small tree of the Solanaceae family. Some of its close relatives are widely cultivated species, such as tomato (*Solanum lycopersicum*), potato (*Solanum tuberosum*), and tobacco (*Nicotiana tabacum*) [1]. Tamarillo is native to the Andean regions of South America (Argentina and Bolivia) and is currently spread worldwide in Central America, Southern Europe (mainly Portugal), and in Oceania [2,3].

The fruits are usually oval measuring 5–10 cm in length and 3–5 cm wide, and their epicarp and pulp can range from red to yellow, a feature that is used to distinguish different cultivars. They appear in groups of 3 to 12 fruits, held by long peduncles. The epicarp is firm with an unpleasant flavor but the pulp is very juicy and more or less bittersweet, depending on the cultivar. The fruit contains a high number of seeds which are flat and round, as in tomato. The fruits have a long ripening season that, in Portugal, can extend from October to April [3,4].

Worldwide, especially in Portugal, there has been a growing interest in tamarillo fruits due to their exceptional nutritional and economic values. They have high amounts of protein, vitamins, and minerals, while being low in carbohydrates and calories [5]. They can be eaten either raw or processed into juices and jams. Several studies have shown that these fruits are rich in phenolic compounds, anthocyanins, and carotenoids with biological and therapeutic importance [6,7,8,9].

FTIR-ATR (Fourier-transform infrared spectroscopy in attenuated total reflectance mode) allows us to analyze the samples and map their constituents. This is a highly accurate, non-destructive method to assess samples’ main components which also allow semi-quantitative comparisons. In a recent publication by Martin et al. (2021) [10], a Raman and FTIR spectroscopic study proved that high amounts of polyphenols are present in tamarillo fruits, particularly in their skin. However, the inner skin and pulp were also shown to contain appreciable amounts of phenols and dietary fibers, evidencing the fruit’s nutraceutical relevance.

Vasco et al. (2009) [11] analyzed the phenolic profile of tamarillo fruits and observed that hydroxycinnamic acids, such as chlorogenic acid (3-caeoylquinic acid) [9], quercetin, and myricetin derivatives were the most abundant. The most commonly found anthocyanins in tamarillo fruits are cyanidin, delphinidin, and pelargonidin glycosides [9]. Due to their content in phenols and anthocyanins, tamarillo has shown a higher antioxidant activity than more commonly eaten fruits, such as apples, oranges, grapes, and tomatoes [8,12], demonstrating the potential of this still poorly explored fruit. 

The production of reactive oxygen species (ROS) can lead to cell damage and interfere with important biomolecules, thus affecting normal cellular function [13]. Phenols, such as flavonoids and anthocyanins, have significant antioxidant properties [14]. They are extremely relevant from a human health point of view as they act as protective agents against free radicals and may therefore be linked to cancer chemoprevention [15], cardiovascular disease risk reduction [16], and even anti-microbial activity [9].

The goal of this study was to evaluate and compare the antioxidant activity of aqueous ethanolic extracts of four genotypes of tamarillo (one imported and three produced in Portugal), following several protocols for antioxidant activity evaluation, aiming at a more complete understanding of their potential. These assays were coupled with an FTIR-ATR analysis of the extracts, in order to determine the chemical composition according to the region of the fruit. This is an innovative approach, coupling analytical and spectroscopic methods for the evaluation of the health-beneficial properties of this fruit relating these activities to its main chemical constituents. In addition, to the best of our knowledge, this is the first such work on tamarillo. These kinds of studies are extremely relevant for public perception of the importance of fruit consumption for human health. The results thus obtained are expected to promote the production and consumption of Portuguese tamarillo genotypes by demonstrating that these are a good source of antioxidant compounds.

## 2. Materials and Methods

### 2.1. Chemicals

2,2′-Azino-bis(3-ethylbenzothiazoline-6-sulphonic acid) diammoniumsalt (≥98%), 2,2′-azobis(2-methylpropionamidine) dihydrochloride (97%), 2,2-diphenyl-1-picrylhydrazyl, 2,4,6-tris(2-pyridyl)-*s*-triazine (≥99%), 5,5′-dithiobis(2-nitrobenzoic acid) (99%), acetylcholinesterase (200–1000 units/mg protein) from *Electrophorus electricus* (electric eel), acetone (≥99%), acetylthiocholine iodide (≥99.0%), aluminum chloride (AlCl_3_, for synthesis), ammonium acetate (≥98%), butylated hydroxytoluene (≥99%), copper(II) chloride (CuCl_2_, for synthesis), ethylenediaminetetraacetic acid (EDTA) (≥98.5%), ethanol absolute, ferrozine (97%), galantamine hydrobromide, gallic acid (≥98%), iron(II) chloride (FeCl_2_·4(H_2_O), ≥99%), linoleic acid (≥99%), methanol (≥99%), neocuproine (≥98%), potassium chloride (KCl) (≥99%), quercetin (≥95%), sodium acetate (≥99%), sodium carbonate (≥99.5%), sodium dihydrogen phosphate (NaH_2_PO_4_·2(H_2_O), ≥98%), thiobarbituric acid (≥98%), trichloroacetic acid, TRIS (≥99%), Trolox (97%), Tween^®^ 80, β-carotene (≥93%), as well as solvents (of analytical grade) were obtained from Merck (Oeiras, Portugal). Acetic acid (glacial p.a.) was purchased from Pronalab (Sintra, Portugal), the Folin–Ciocalteu’s reagent, HCl (35%) and iron (III) chloride (FeCl_3_∙6(H_2_O, ≥98%)) from Panreac (Barcelona, Spain), and potassium persulfate (99%) and sodium phosphate dibasic (Na_2_HPO_4_, ≥99%) from Honeywell (Carnaxide, Portugal).

### 2.2. Biological Material

Five randomly selected mature fruits were collected from four different genotypes of tamarillo. Red tamarillos (TVM) collected in Mealhada (Portugal), red (CR) and orange (CO) tamarillos from the Botanical Garden of the University of Coimbra (Portugal), and imported red tamarillos from Colombia bought in a local supermarket (TMI). Each fruit was freeze-dried and ground into a fine powder.

### 2.3. Physical Properties

Five fruits were weighed and their diameter, length, and peduncle were measured for each genotype. Their hardness was determined by a Precision Hardness Sclerometer (Lutron Electronic, Fr—5120, Hanoi city, Vietnam). The soluble solid content was determined by a Digital Refractometer (Kern^®^ optics, ORD 85BM, Frankfurt am Main, Germany). Fruit acidity was measured by an acidity meter (Atago Co., Ltd., Pal-Easy Acid 12 Kit, Tokyo, Japan) using squeezed fruit juice. For each property the mean was calculated.

### 2.4. Aqueous Ethanolic Extraction

Aqueous ethanolic extracts were produced from the tamarillo fruits of each set of samples. Briefly, 30 mL of aqueous ethanol 70% (*v*/*v*) was added to 1 g of accurately weighed previously freeze-dried and ground fruits. To maximize the extraction, this mix was left macerating for 12 h, overnight, in a shaking incubator set to 25 °C/130 rpm. The samples were then centrifuged (900× *g*; 10 min) and the supernatant was collected. Subsequently, 2 further extraction steps were performed on the solid residues, both with 30 mL of aqueous ethanol 70% (*v/v*) and with a maceration time of 30 min, in a shaking incubator set to 25 °C/130 rpm. After each step, the supernatants were collected upon centrifugation (900× *g*; 10 min) and filtered through Whatman 70 mm filter paper. Finally, the supernatants resulting from these three extraction steps were combined. Extract concentrations were determined as mass of dry extract per mL of extract (mg mL^−1^): 1 mL of the extract was transferred to a tube and left to dry at 40 °C for 48 h (experiment performed in triplicate). Extract stock solutions were kept in the dark, at 4 °C, until subsequent analysis.

### 2.5. Antioxidant Properties

Aqueous ethanolic extracts from each tamarillo genotype were used for the antioxidant analyses. For each assay, three independent experiments were performed. The samples were measured in a multi-well spectrophotometer (µQuant™ Microplate spectrophotometer, BioTek Instruments Inc., Bad Friedrichshall, Germany). The blanks consisted of a mixture of the reagent’s solvents. The protocols followed to assess the antioxidant capacity were the same as described in Marques et al. (2021) [17].

#### 2.5.1. Total Phenolic Compounds (TPC), Total Flavonoids Content (TFC), and Total Monomeric Anthocyanin Content (TMAC)

The TPC of the four extracts was determined using the Folin–Ciocalteu method [18]. As a reference standard, gallic acid was used (2.5–50 μg mL^−1^ concentration range). The results were expressed as mg of gallic acid equivalents (GAE) per gram of dry weight (DW).

The TFC of the extracts was determined by the aluminum chloride (AlCl_3_) method [19]. As a reference standard, quercetin was used (7.5–75 μg mL^−1^ concentration range). The results were expressed as mg of quercetin equivalents (QCTE) per gram of extract. 

The total monomeric anthocyanin content was determined by the pH differential method [20]. Results were expressed as mg of cyanidin-3-glucoside equivalents (C3GE) per dry weight.

#### 2.5.2. 2,2-Diphenyl-1-picrylhydrazyl (DPPH) and 2,2′-Azino-bis (3-ethylbenzothiazoline-6-sulfonic Acid) (ABTS)

The scavenging ability of the DPPH radical was determined [21]. Trolox was chosen as a reference antioxidant, and for the control a DPPH solution diluted with methanol was used. The results were expressed as IC_50_ values (mg mL^−1^) calculated using nonlinear regression analysis.

The scavenging of the ABTS free radical was determined [22]. Trolox was used as a reference antioxidant. For the control, an ABTS radical solution diluted with ethanol was used. The results were expressed as IC_50_ values (mg mL^−1^) calculated using nonlinear regression analysis.

#### 2.5.3. β-Carotene–Linoleic Acid Bleaching Method and Inhibition of Lipid Peroxidation in Buffered Egg Yolk

The inhibition of the coupled oxidation of the linoleic acid/β-carotene system was assessed [23]. Butylated hydroxytoluene (BHT) was used as a reference antioxidant. For the control, emulsion with ethanol was used. The results were expressed as IC_50_ values (mg mL^−1^) calculated using nonlinear regression analysis.

The inhibition of the formation of thiobarbituric acid reactive substances (TBARS) was evaluated [24]. The results were expressed as IC_50_ values (mg mL^−1^) calculated using nonlinear regression analysis.

#### 2.5.4. Metal Chelating Ability, Ferric Reducing Antioxidant Power Assay (FRAP), and Cupric Ion Reducing Antioxidant Capacity Assay (CUPRAC)

The iron-chelating activity was measured as previously reported [25]. As a reference standard, EDTA was used (3.125–50 μg mL^−1^ concentration range). The results were expressed as mg of EDTA equivalents per gram of dry weight. 

FRAP [26] and CUPRAC [27] tests were applied. Trolox (16–250 μg mL^−1^) was used as a reference standard. The results were expressed as mg of Trolox equivalents (TE) per gram of dry weight.

#### 2.5.5. Enzymatic Activity (Cholinesterase Inhibition)

The inhibitory activity of the acetylcholinesterase (AChE) enzyme was determined [28]. As a reference standard, galantamine was used (6.125–25 μg mL^−1^ concentration range). The results were expressed as mg galantamine equivalents (GALAE) per gram of dry weight.

### 2.6. Fourier-Transform Infrared Spectroscopy

FTIR spectra were obtained for the freeze-dried aqueous ethanol extracts. Triplicates of each spectrum were acquired in the range of 4000–600 cm^−1^ using a Vertex 70 FTIR spectrometer (Bruker Optics, Ettlingen, Germany) purged by CO_2_-free dry air and equipped with a Brucker Platinum ATR single-reflection diamond accessory. A Ge on KBr substrate beamsplitter with liquid nitrogen-cooled wide band mercury cadmium telluride (MCT) detector was used. Spectra were averaged over 32 scans at a resolution of 4 cm^−1^, and the 3-term Blackman–Harris apodization function was applied. The integrated Bruker Opus 8.1 software was used to compensate H_2_O and CO_2_ contributions. The spectra were imported into MatLab (v. R2021b; MathWorks, Natick, MA, USA). Using the Eigenvector PLS Toolbox (v. 9.0; Eigenvector Research, Wenatchee, WA, USA), the full spectra were vector normalized to unit length (2-Norm), the baseline was removed according to the automatic weighted least squares algorithm (polynomial order = 2), and the extended multiplicative scatter correction (EMSC) was applied. Then, the spectra were restricted to the fingerprint region (1800–800 cm^−1^). The data were handled using the Origin software (version 2020 9.7, Origin Software Solutions, Irvine, CA, USA).

### 2.7. Statistical Analysis

The results from the physical properties and antioxidant assays were analyzed in GraphPad Prism (version 9, Dotmatics, Boston, MA, USA) using one-way ANOVA followed by Tukey’s post hoc test for statistical comparison between each extract, *p*-values less than or equal to 0.05 were considered as significant, and these differences were represented by different superscript letters in a row of results. IC_50_ values were calculated using nonlinear regression analysis, in sigmoidal dose–response curves (variable slope).

## 3. Results

### 3.1. Physical Properties

**Figure 1:**
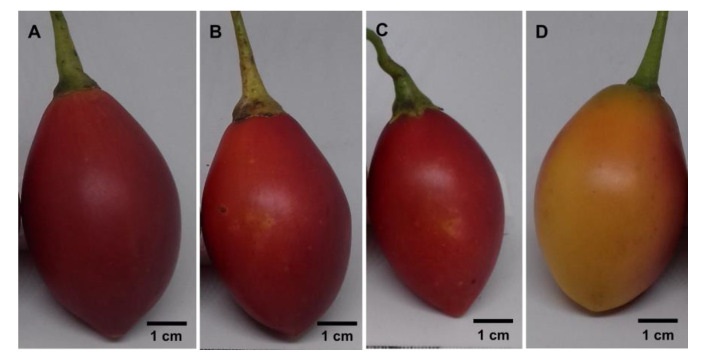
Tamarillo fruits: (**A**) TMI (red) from Colombia; (**B**) TVM (red) from Mealhada; (**C**) CR (red) from the Botanical Garden of Coimbra; (**D**) CO (orange) from the Botanical Garden of Coimbra.

This work aims to highlight tamarillo fruits for processing and human consumption. All analyzed fruits were in the same stage of ripeness. TMI (Figure 1A) fruits were bulkier, with a lower soluble solid content (SSC) (Table 1). The other red fruits, TVM (Figure 1B) and CR (Figure 1C), were very similar in shape, size, and SSC but CR was more acidic. The orange fruits, CO (Figure 1D), were similar in shape, size, SSC, and acidity to TVM and CR.

### 3.2. Total Phenolic, Flavonoid, and Monomeric Anthocyanin Content

The TVM extract showed higher TPC followed by CR while CO and TMI presented the lowest values (Table 2).

The results showed that the CR and CO extracts had the highest value of total TFC followed by TVM. TMI had the lowest concentration values. 

Regarding the TMAC, the red fruits TMI, TVM, and CR had a higher content of anthocyanins (these pigments are responsible for the red color in these fruits). CO, the orange fruits, had a value of TMAC close to 0.

### 3.3. DPPH and ABTS 

The red fruits showed a higher radical scavenging ability both towards the DPPH radical and the ABTS radical cation (Table 3). TVM showed the highest activity, followed by TMI and CR. CO had a significantly lower antioxidant capacity to reduce both radicals. Unfortunately, none of the extracts were able to reach the antioxidant activity of the standard Trolox, which yielded much lower IC_50_ values (Table 4).

### 3.4. β-Carotene Oxidation Inhibition and Lipid Peroxidation Inhibition in Buffered Egg Yolk

According to the β-carotene–linoleic acid bleaching assay, TMI and CO showed the highest inhibitory capacity of β-carotene oxidation (Table 3). Nevertheless, they are still considerably less active than BHT (IC_50_ = 0.125 ± 0.015 mg mL^−1^) (Table 4). On the other hand, the other TVM and CR red genotypes yielded the lowest capacities.

The extracts’ ability to inhibit lipid peroxidation was found to be very similar (Table 3), with IC_50_ values not differing statistically and much higher than the IC_50_ value reported for the standard antioxidant BHT (Table 4).

### 3.5. Metal Chelating Ability, FRAP, and CUPRAC

Regarding the metal ion chelating ability (Table 3), TVM showed a higher capacity to disrupt the Fe^2+^–ferrozine complex, followed by CO. CR and TMI had the lowest capacity.

The extracts’ ability to reduce copper and iron followed the same trend, with TMI presenting the lowest value of TE mg g^−1^ DW, followed by CO, and with TVM and CR having the highest values.

### 3.6. Enzymatic Activity (AChE Inhibition)

The ability to inhibit AChE (Table 3) was higher for the TVM extract, followed by CO and CR, while TMI showed the lower inhibitory capacity towards this enzyme.

### 3.7. Spectroscopic Analysis

**Figure 2:**
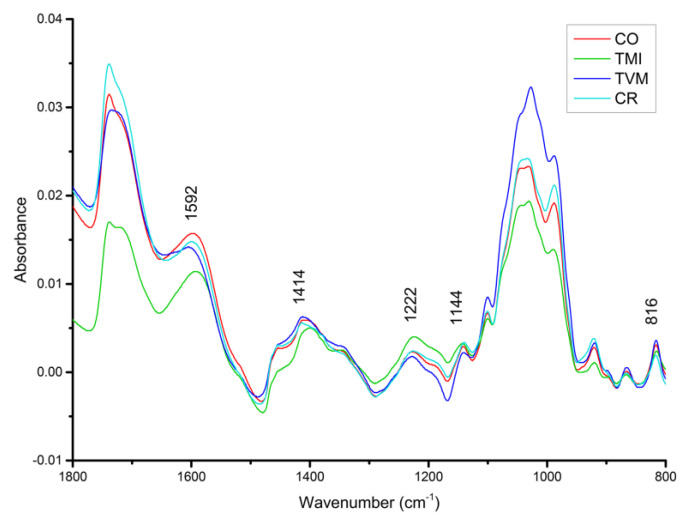
Mean FTIR spectra (1800–800 cm^−1^) of the tamarillo extracts presently studied. TMI, imported red tamarillo; TVM, Mealhada red tamarillo; CR, Botanical Garden red tamarillo; CO, Botanical Garden orange tamarillo.

The FTIR-ATR spectra were obtained from freeze-dried aqueous ethanolic extracts (Figure 2). The spectra were examined in the fingerprint region (1800–800 cm^−1^), and assignments were made for each band of interest (Table 5). The aqueous ethanolic extracts were rich in phenolic compounds (816 cm^−1^; 1144 cm^−1^; 1222 cm^−1^; 1414 cm^−1^; 1592 cm^−1^) and polysaccharides (988 cm^−1^; 1045 cm^−1^; 1222 cm^−1^; 1720/1739 cm^−1^), namely, pectins (1032 cm^−1^) and esters (1100 cm^−1^). The spectra suggest the presence of triterpenoids (922 cm^−1^) and cutin/waxes (1346 cm^−1^). Two bands at 865 cm^−1^ (C-H out-of-plane) and at 1454 cm^−1^ (δ(CH_2_)_scissoring_), due to polysaccharides, were also observed.

Since the phenolic compounds should be the main compounds responsible for the extracts´ antioxidant activity, the subsequent analysis will focus only on the bands assigned to these constituents (Figure 2) by comparing the relative intensity of these bands (semiquantitative approximation). For each spectral region of interest, the genotypes were classified from higher to lower intensity of the bands (Figure 2) as follows: 1592 cm^−1^—CO, CR, TVM, TMI; 1414 cm^−1^—TVM, CO, CR, TMI; 1222 cm^−1^—TMI, CR, CO, TVM; 1144 cm^−1^—CR, TMI, CO, TVM; 816 cm^−1^—TVM, CO, TMI, CR.

## 4. Discussion

Tamarillo is still a poorly known but exciting fruit, with a high nutraceutical and economic potential, not only for the fruit itself but also for processed products, such as juices, jams, and yogurts [5]. Most studies on the tamarillo antioxidant potential were performed in the subparts of the fruit but not for extracts prepared from the fruit as a whole. Hence, this study aimed to provide an insight into the overall potential of the fruit. Different assays were performed with a view to determine the antioxidant potential of different genotypes of tamarillo. Previous works characterized some chemical components present in the fruits such as phenols, polyphenols, and others, with antioxidant capacity [5,8,9,10,11]. Presently, for a comparative analysis, BHT and Trolox were used as reference antioxidants as shown in Table 4. From previous studies, several antioxidant potential compounds from tamarillo were analyzed, such as phenolics and anthocyanins [5].

### 4.1. Physical Properties

In terms of the physical comparison (Table 1), the imported fruits differed from the Portuguese ones regarding their size, with a lower concentration of SSC. The Portuguese genotypes, in turn, were very similar between them and had a higher concentration of SSC.

### 4.2. Total Phenolic Compounds (TPC), Flavonoids (TFC), and Anthocyanins (TMAC)

Plant phenolic content is positively linked to antioxidant activity [14]. The total phenolic content determined for the tamarillo extracts currently analyzed was found to be higher for TVM and CR (3.47 mg and 3.17 GAE mg g^−1^ DW) when compared with the imported (TMI—1.82 GAE mg g^−1^ DW) and orange (CO—2.52 GAE mg g^−1^ DW) genotypes. These results are in agreement with the TPC values reported for red tamarillos, varying from 2.53 to 7.63 GAE mg g^−1^ DW [8,12,47], and for 3 cultivars of tomato (4.25–5.07 GAE mg g^−1^ DW [12] and eggplant (*Solanum melongena* L.) (3.59–6.54 GAE mg g^−1^ DW) [48] (both from the same family as tamarillo), as well as for grapes (*Vitis vinifera* L.), a popular source of antioxidants (3.59–6.54 GAE mg g^−1^ DW) [49]. Several studies have shown a correlation between high contents of phenols and the antioxidant potential in tamarillo [12,47] and in its relative, the tomato [50], and between flavonoids and antioxidant activity [12].

Flavonoids are also directly correlated to antioxidant activity and free radical scavenging ability. The amount of flavonoids found in the presently studied samples are likely due to the presence of quercetin and myricetin [11]. The total flavonoid content was similar between TMI and TVM (3.39 and 4.20 QCTE mg g^−1^ DW), as well as between CR and CO (5.55 and 5.33 QCTE mg g^−1^ DW), with the Portuguese genotypes showing higher amounts. These genotypes (CR and CO) showed similar or higher flavonoid contents than those reported elsewhere for other tamarillos (6.44 and 3.2 QCTE mg g^−1^ DW) [12,47], lower than in eggplant (7.17–14.24 QCTE mg g^−1^ DW) [48], and higher than in tomato (1.66 QCTE mg g^−1^ DW) and yellow and red cherry tomatoes (1.88 and 1.71 QCTE mg g^−1^ DW) [12].

The presence of anthocyanins in tamarillo has been reported by Diep et al. (2020) [9], who have identified cyanidin, delphinidin, and pelargonidin glycosides. In Portuguese tamarillo, peonidin-3-*O*-glucoside was the most abundant anthocyanin [51]. Anthocyanins are known for their antioxidant potential and are present mainly in the Portuguese genotypes TVM and CR (0.62 and 0.33 C3GE mg g^−1^ DW, respectively) when compared with TMI and CO (0.18 and 0.11 C3GE mg g^−1^ DW, respectively). These quantities fall in the same range as reported by Niño-Medina et al. (2014) [52] in eggplant, where the TMAC values were as low as 0.039–1.61 C3GE mg g^−1^ DW.

### 4.3. Antioxidant and Enzymatic Activities

DPPH and ABTS are commonly used assays to determine antioxidant capacity. IC_50_ values were obtained for each sample, with lower values corresponding to a higher antioxidant potential. Vasco et al. (2009) [11] showed a correlation between the TPC and DPPH assays. The current results also demonstrate a similar correlation, although not statistically significant. The genotypes with the highest overall content of phenols and flavonoids, TVM (best), TMI, and CR, showed a lower IC_50_ which translates into a stronger antioxidant potential. Other studies also evidenced a high antioxidant potential in tamarillo fruits (based on the DPPH assay), with low IC_50_ values—0.80 and 0.089 mg mL^−1^ [6,47]—which is higher than those obtained for our tamarillo samples (Table 3). The ABTS assay yields data that follow the same trend. Espín et al. (2016) [53] showed that the red tamarillo fruits had a higher antioxidant activity than their non-red counterparts, corroborating our higher results obtained for the red genotypes (TVM, TMI, and CR) when compared with the orange one (CO).

To analyze the inhibition of the linoleic acid/β-carotene oxidation system, the β-carotene–linoleic acid bleaching assay was performed. The goal was to analyze which tamarillo genotype displayed the highest inhibitory capacity regarding the oxidation of β-carotene that results from ROS produced by linoleic acid oxidation. It was possible to reach an inhibition of at least 50%, contrary to what was previously reported [12]. Surprisingly, the CO and TMI extracts, with the lowest antioxidant potential (determined by the DPPH and ABTS methods), showed a higher capacity according to the β-carotene–linoleic acid bleaching method. Mutalib et al. (2017) [47] showed that the tamarillo extracts presented a high percentage of inhibition (79.30%), although still far from the antioxidant of reference, in accordance with the present results (BHT—95.60%).

The FRAP and CUPRAC assays were used to measure the potential of the antioxidants present in the extracts to reduce the Fe(III)–TPTZ and Cu(II)-neocuproine complexes, respectively. Diep et al. (2020) [9] showed a correlation between CUPRAC, FRAP, and TPC, which was similar to the trend revealed by the current results: higher activity measured for CUPRAC and FRAP, corresponding to the highest TPCs. TVM, the sample with the highest TPC, showed the highest significative activity for FRAP and CUPRAC assays, while TMI, the imported fruit, had the lowest activity of all samples.

The metal chelating activity assay (chelation of Fe^2+^, a metal ion involved in the formation of ROS) determines the capacity of a sample to disrupt the metallic complex between Fe^2+^ and ferrozine. This disruption is due to the coordination of phenols to the iron ion, substituting ferrozine. The trend observed for the metal chelating assay was similar to that found for the FRAP and CUPRAC assays. The phenolic compounds quercetin and rutin, present in tamarillo [9], are very effective Fe^3+^ and Cu^2+^ chelating agents [17]. The FRAP results obtained for the different tamarillo genotypes clearly evidenced a higher potential of the Portuguese red genotypes when compared with the Portuguese orange ones. This is in accordance with previous reports using different colored fruits (red vs. yellow) that presented values of 15–50 µmol Trolox/g dry fruit for red genotypes and 10–17 µmol Trolox/g dry fruit for yellow ones [53], and of 60–161 µmol Trolox/g dry fruit for red tamarillo and 52–85 µmol Trolox/g dry fruit for yellow [9]. For the CUPRAC assay, similar results had also been reported by Diep et al. (2020) [9]: yellow—43–118 µmol Trolox/g dry fruit; red—52–265 µmol Trolox/g dry fruit.

Lipid peroxidation is an oxidative process that results in lipid degradation and ultimately in cell damage. Malondialdehyde (MDA), a marker of lipid peroxidation, reacts with thiobarbituric acid (TBA) to generate an MDA-TBA complex, allowing for its quantification. In the present study, no significant differences were detected regarding lipid peroxidation inhibition for the distinct tamarillo genotypes analyzed, which can mean that relevant polyphenols for lipid peroxidation inhibition are present in identical amounts in the different fruit genotypes. However, it has been reported that tamarillo peels were able to delay lipid oxidation in cooked beef during storage [54]. Thus, more studies on this subject might reveal the true potential of tamarillo phytochemicals in lipid peroxidation prevention.

Analysis of the inhibitory activity of the samples towards AChE was performed by the Ellman method. AChE is responsible for the hydrolysis of acetylcholine (ACh), an important compound in neurotransmission (associated with learning and memory functions), thus the use of AChE inhibitors is one of the currently available treatment options for Alzheimer’s disease [55]. Studies have been reported on the neuroprotective potential of tamarillo epicarp, which may be due to this type of mechanism [56]. The tamarillo samples under study, however, showed no significant activity regarding AChE inhibition.

As to the overall antioxidant performance of each fruit, the TVM (red) tamarillo presents the highest antioxidant potential, followed by the CR (red), the CO (orange), and the TMI (red) genotypes.

### 4.4. Spectroscopic Analysis 

FTIR-ATR enabled the attainment of a highly sensitive and specific characterization of functional groups in the different extracts, corresponding to their main chemical constituents. Freeze-dried aqueous ethanolic extracts from all tamarillo genotypes were analyzed. Spectral regions of the resulting spectroscopic data were assigned to their respective chemical functional groups, which may be correlated to the fruit’s antioxidant potential.

All extracts were found to be rich in phenolic compounds (Figure 2), which are key molecules for antioxidant activity. Their main infrared signals were detected: 1592 cm^−1^, 1414 cm^−1^, 1222 cm^−1^, and 816 cm^−1^ (see Table 5 for details). Furthermore, bands ascribed to polysaccharides were also detected: 1739 cm^−1^, 1720 cm^−1^, 1222 cm^−1^, 1100 cm^−1^, 1045 cm^−1^, 1032 cm^−1^, and 988 cm^−1^.

For the purpose of this study, the most important compositional trait of the extracts is their phenolic content. Indeed, the antioxidant activities detected for tamarillo fruit extracts are primarily attributed to these compounds [14,57], owing to their action as reducing agents (hydrogen donors), singlet oxygen quenchers, and/or metal chelators [58]. Accordingly, as FTIR-ATR enabled the detection of functional groups associated with phenolic compounds, it is plausible that the presence of these molecules in the extracts is directly associated with their antioxidant activity [59,60].

Attending to the data comprised in Table 2, TMI has the lowest value for TPC (phenols), which is also evidenced by the very low intensity of the peak at 1592 cm^−1^ (phenols) when compared with the others (Figure 2). These results highlight that TMI has lower amounts of phenols, which explains its lower antioxidant capacity [12]. TVM shows the highest TPC and TFC activities which is consistent with its infrared profiles for phenols similar to those from CO and CR (Figure 2), although there is a small difference in their TFC (CR has a higher phenolic content than CO). This may explain why TVM has the highest antioxidant activity. However, CR was found to have a much higher antioxidant activity when compared with CO, which performs similarly to TMI (lowest amount of phenols). This can be justified by the low amount of anthocyanins that were measured for CO (TMAC, Table 2), as these are also relevant components for antioxidant capacity [14].

These antioxidant properties are responsible for tamarillo’s health-beneficial properties, which may render it an interesting nutraceutical compound. Nonetheless, in plants, the role of phenols is tightly linked to responses to biotic and abiotic stresses, as well as to developmental or physiological factors [61]. Therefore, the variations observed in the relative antioxidant activity and abundance of phenolic compounds between the tested samples can originate from distinct physiological adaptive strategies, which may be genotype dependent. Indeed, it has been reported that in some fruits, such as apples, phenolic contents are strongly dependent on the fruit cultivar [62]. This may provide a justification for the distinct properties currently found for the two different tamarillo genotypes grown in similar conditions—CR and CO, from the Botanical Garden of the University of Coimbra (Portugal). For the TMI and TVM genotypes, respectively imported from Colombia and grown in Mealhada (Portugal), in addition to genotype differences, other factors might be at play, such as distinct edaphoclimatic settings, agricultural practices, or storage conditions, which may vary between locations and are known to significantly affect phenolic concentrations [9,62]. Particularly for the TMI genotype (Colombia) when compared with locally sourced fruits, it cannot be excluded that transportation time and conditions may have had some effect on the fruit properties. In fact, whereas the Portuguese genotypes were analyzed immediately after harvesting, for the fruits from Colombia the transport and shelf times must be considered. This is particularly relevant if we consider that fruit antioxidants are highly prone to degradation under certain conditions such as chilling injury, irradiation, fungal decay, or inadequate handling practices (e.g., temperature and relative humidity) [63].

Triterpenoids, known for their anti-inflammatory and anti-cancer activities, are another class of fruit constituents (main band at 922 cm^−1^), and are usually present in the external part of the skin protecting the pulp from deleterious radiation and weather conditions [64].

## 5. Conclusions

Tamarillo has a high potential to be developed as a high-profile fruit worldwide due to its health-beneficial and nutritious properties and significant antioxidant activity. Therefore, it can become a good alternative to other, more common, edible fruits. The detection of phenols by FTIR-ATR and the total phenol content measured for fruit samples of different genotypes and sources showed that tamarillo is rich in these compounds, which are the main contributors to the fruit´s high antioxidant potential. The red Portuguese genotypes, mainly Mealhada red tamarillo (TVM), were found to contain particularly large phenol contents and consequently a high antioxidant activity. Therefore, tamarillo should be considered by farmers and distributors as a promising product for nutraceutical development.

## Figures and Tables

**Table 1:** Physical properties of the tamarillo genotypes of the present study.

Assay/Extract	TMI	TVM	CR	CO
Weight (g)	94.41 ± 11.29 ^a^	57.16 ± 5.84 ^b^	47.36 ± 4.01 ^b^	57.71 ± 7.34 ^b^
Fruit diameter (cm)	5.17 ± 0.26 ^a^	4.24 ± 0.19 ^b^	3.86 ± 0.10 ^b^	4.29 ± 0.21 ^c^
Fruit length (cm)	6.46 ± 0.45 ^a,b^	6.79 ± 0.15 ^b^	6.65 ± 0.34 ^a,b^	6.25 ± 0.28 ^a^
Fruit + peduncle length (cm)	10.99 ± 0.69 ^a,b^	11.49 ± 0.30 ^b^	11.01 ± 0.46 ^a,b^	10.51 ± 0.62 ^a^
Peduncle diameter (cm)	0.74 ± 0.14 ^a^	0.70 ± 0.04 ^a^	0.68 ± 0.05 ^a^	0.63 ± 0.06 ^a^
Hardness (N/cm^2^)	17.46 ± 1.18 ^a^	18.14 ± 1.17 ^a^	15.30 ± 2.35 ^a^	17.55 ± 1.86 ^a^
Soluble solid content (°Brix)	8.17 ± 0.92 ^c^	10.35 ± 0.24 ^a,b^	11.28 ± 0.46 ^a^	9.77 ± 0.12 ^b^
Acidity (% of total acidity to citric acid conversion)	1.83 ± 0.13 ^c^	1.86 ± 0.05 ^b,c^	2.32 ± 0.15 ^a^	2.04 ± 0.08 ^b^

TMI, imported red tamarillo; TVM, Mealhada red tamarillo; CR, Botanical Garden red tamarillo; CO, Botanical Garden orange tamarillo. Values represent the mean ± standard deviation of three independent experiments. For the same row, different superscript letters indicate significant differences (Tukey’s post hoc test, *p* ≤ 0.05).

**Table 2:** Chemical composition of the tamarillo extracts of the present study (TPC, TFC, and TMAC).

Assay/Extract	TMI	TVM	CR	CO
TPC (GAE mg g^−1^ DW)	1.82 ± 0.05 ^c^	3.47 ± 0.18 ^a^	3.17 ± 0.11 ^a^	2.52 ± 0.06 ^b^
TFC (QCTE mg g^−1^ DW)	3.39 ± 0.11 ^b^	4.20 ± 0.37 ^b^	5.55 ± 0.43 ^a^	5.33 ± 0.33 ^a^
TMAC (C3GE mg g^−1^ DW)	0.18 ± 0.05 ^b,c^	0.62 ± 0.05 ^a^	0.33 ± 0.05 ^b^	0.11 ± 0.04 ^d^

TMI, imported red tamarillo; TVM, Mealhada red tamarillo; CR, Botanical Garden red tamarillo; CO, Botanical Garden orange tamarillo; GAE, gallic acid equivalents; DW, dry weight; QCTE, quercetin equivalents; C3GE, cyanidin-3-glucoside equivalents. Values represent the mean ± standard deviation of three independent experiments. For the same row, different superscript letters indicate significant differences (Tukey’s post hoc test, *p* ≤ 0.05).

**Table 3:** Antioxidant and enzymatic activities of the different tamarillo genotypes presently studied.

Assay/Extract	TMI	TVM	CR	CO
DPPH (IC_50_ mg mL^−1^)	1.79 ± 0.08 ^c^	1.33 ± 0.04 ^d^	2.03 ± 0.08 ^b^	3.34 ± 0.08 ^a^
ABTS (IC_50_ mg mL^−1^)	5.89 ± 0.02 ^a,b^	3.70 ± 0.04 ^c^	5.62 ± 0.09 ^b^	10.17 ± 0.17 ^d^
β-carotene/linoleic acid (IC_50_ mg mL^−1^)	3.69 ± 0.37 ^b,c^	5.20 ± 0.33 ^a^	4.62 ± 0.33 ^a,c^	3.79 ± 0.24 ^c^
Lipid peroxidation (IC_50_ mg mL^−1^)	3.38 ± 0.51 ^a^	3.22 ± 0.57 ^a^	3.06 ± 0.63 ^a^	3.23 ± 0.27 ^a^
Metal chelating ability (EDTA mg g^−1^ DW)	1.22 ± 0.04 ^c^	2.17 ± 0.11 ^a^	1.71 ± 0.02 ^b^	1.98 ± 0.04 ^a^
FRAP (TE mg g^−1^ DW)	2.56 ± 0.06 ^d^	5.67 ± 0.17 ^a^	4.74 ± 0.07 ^b^	2.96 ± 0.06 ^c^
CUPRAC (TE mg g^−1^ DW)	5.26 ± 0.07 ^c^	11.11 ± 0.35 ^a^	10.54 ± 0.19 ^a^	7.53 ± 0.04 ^b^
AChE (GALAE mg g^−1^ DW)	0.10 ± 0.02 ^c^	0.95 ± 0.10 ^a^	0.44 ± 0.05 ^b^	0.49 ± 0.12 ^b^

TMI, imported red tamarillo; TVM, Mealhada red tamarillo; CR, Botanical Garden red tamarillo; CO, Botanical Garden orange tamarillo; DW, dry weight; TE, Trolox Equivalents; GALAE, galantamine equivalents. Values represent the mean ± standard deviation of three independent experiments. For the same row, different superscript letters indicate significant differences (Tukey’s post hoc test, *p* ≤ 0.05).

**Table 4:** Antioxidant activity of Trolox and BHT standards.

Assay/Reference Antioxidant	Trolox	BHT
DPPH (IC_50_ mg mL^−1^)	0.065 ± 0.003	–
ABTS (IC_50_ mg mL^−1^)	0.084 ± 0.001	–
β-carotene/linoleic acid (IC_50_ mg mL^−1^)	–	0.125 ± 0.015
Lipid peroxidation (IC_50_ mg mL^−1^)	–	0.009 ± 0.005 [17]

**Table 5:** FTIR-ATR wavenumbers (cm^−1^) and tentative assignments for the tamarillo extracts under study.

Wavenumber (cm^−1^)	Reference	Assignment ^a^	Compositional Feature
1739/1720	1735 [29] 1739 [30] 1734 [31] 1735 [10]	ν(C=O)	Polysaccharides
1592	1595 [30] 1594 [32] 1595 [33]	Aromatic ring vibration and ν(C=O)	Phenolics
1454	1455 [10] 1456 [34] 1457 [35]	δ(CH_2_) scissoring	Polysaccharides
1414	1419 [36] 1419 [37]	Aromatic skeletal vibration combined with CH in plane deformation	Phenolics
1346	1344 [35] 1344 [38]	δ(CH_2_) wagging and twisting	Cutin, waxes
1222	1220 [29] 1226 [39]	CC, CO, C=O stretches	Polysaccharides and phenolics
1144	1142 [39] 1140 [40] 1136 [41]	aromatic CH in-plane deformation	Phenolics
1100	1098, 1105 [10] 1090 [42] 1103 [38]	ν(C-O-C)ester	Polysaccharides (esters)
1045	1047 [42] 1054 [35] 1052 [10]	ν(C-O-C)glycosidic	Polysaccharides
1032	1032 [42] 1034 [10] 1035 [43] 1030 [44]	ν(C-O) and ν(C-C)	Polysaccharides (pectins)
988	993 [45] 995 [10] 985 [36]	ν(CO) and ν(CC)	Polysaccharides
922	918 [42]	ρ(CH_3_)	Triterpenoids
865	866 [40] 869 [41]	C-H out-of-plane	-
816	816 [35] 810 [46]	Aromatic C-H out-of-plane deformations	Phenolics

^a^ ν, stretching; δ, bending; ρ, rocking.

## Data Availability

The data presented in this study are available in the article.
